# Intravesical Therapies for Recurrent Urinary Tract Infections: A Systematic Review

**DOI:** 10.7759/cureus.72175

**Published:** 2024-10-23

**Authors:** Dhruv Patel

**Affiliations:** 1 Department of Urology, Gloucestershire Hospitals NHS Foundation Trust, Cheltenham, GBR

**Keywords:** chondroitin sulphate, cystitis, intravesical instillation, intravesical therapy, recurrent uti, through-catheter antibiotics, hyaluronic acid

## Abstract

Recurrent urinary tract infections (rUTIs) present a significant clinical challenge, particularly due to the associated overuse of antibiotics and the rise in antimicrobial resistance. This systematic review evaluates the current literature on the use of intravesical therapies as an alternative treatment for rUTIs. Two established primary therapies are reviewed: glycosaminoglycan (GAG) instillations and intravesical antibiotic instillations. Both therapies offer localised treatment, reducing systemic antibiotic exposure and targeting infection sites more directly.

A literature search was conducted using PubMed and Cochrane Controlled Register of Trials (CENTRAL), yielding 5,963 relevant articles, of which seven studies met the inclusion criteria. The review indicates that both GAG and antibiotic instillations significantly reduce UTI recurrence rates and improve symptoms such as pain and urinary urgency. However, significant variations in treatment schedules and dosages exist, and no direct comparative studies between GAG instillations and intravesical antibiotics were found. Moreover, intravesical antibiotics show great potential in minimising antimicrobial resistance, though further large-scale studies are needed to confirm these findings.

While intravesical therapies are generally well-tolerated, GAG instillations can cause mild irritation. Further research is required to optimise therapy regimens and to perform cost-benefit analyses, particularly considering the high costs of these therapies compared to traditional antibiotic prophylaxis. Randomised controlled trials comparing different intravesical treatments are crucial to inform future clinical practice.

## Introduction and background

Recurrent urinary tract infections (rUTIs) represent a significant clinical challenge for urologists, microbiologists, and the healthcare system at large, while greatly impacting patients' quality of life. Urinary tract infections are one of the most common bacterial infections, with a substantial proportion of patients, particularly women, experiencing recurrent episodes despite initial treatment. Recurrent urinary tract infections are defined by the occurrence of at least two episodes in six months or three within a year [1]. Effective management often requires a multidisciplinary approach, and the increasing use of intravesical therapies is being observed [2].

For urologists, the recurrent nature of these infections often means exploring beyond standard antibiotic regimens, with consideration given to anatomical abnormalities, dysfunctional voiding patterns, or immune deficiencies. Recurrent urinary tract infections can be particularly frustrating to manage, as they may involve multi-drug-resistant organisms and lack a universally effective treatment strategy [2]. Intravesical therapies, which involve delivering treatments directly into the bladder, offer a promising alternative to systemic antibiotics by targeting the site of infection more effectively [3]. However, their application and efficacy remain an area of ongoing research and associated clinical trials.

Patients dealing with rUTIs face substantial physical discomfort, emotional distress, and disruption to daily life. Additionally, the frequent use of antibiotics can lead to gastrointestinal disturbances and allergic reactions, further complicating their care. Patients often endure recurrent pain, urinary urgency, and fear of relapse, which can severely impair their quality of life [4]. Intravesical therapies present a potential solution by reducing infection frequency and offering a direct means of eradicating uropathogens while minimising systemic side effects.

Recurrent urinary tract infections are known to contribute to the rise of antimicrobial resistance, and this further complicates their management as standard treatment regimens become less effective [5]. Intravesical therapies offer a localised treatment approach, which may help limit the development of resistance by reducing systemic antibiotic exposure. However, the variability in pathogen strains, biofilm formation, and bladder microenvironments requires ongoing microbiological research to optimise these therapies [6].

From a healthcare system perspective, rUTIs contribute to significant resource use, including frequent medical consultations, diagnostic tests, and hospitalisations for severe cases [1]. Intravesical therapies, if proven effective, could alleviate some of these pressures by reducing the need for repeated courses of antibiotics and reducing hospitalisations; however, their integration into clinical practice requires consideration of cost, accessibility, and patient education.

Despite the huge benefits intravesical therapies could offer, there is little evidence to suggest which type of therapy is more beneficial for patients in terms of improving UTI rates but taking into account tolerability and adherence [2]. This systematic review aims to evaluate the current literature on intravesical treatments for rUTIs and provides an overview of outcomes for the two commonly used types of intravesical therapy to further guide clinical practice.

The two commonest forms of intravesical therapy for rUTIs are glycosaminoglycan (GAG) instillation and intravesical antibiotic instillation [7]. On the horizon, there are further therapies being developed, such as antimicrobial-loaded nanoparticles and intravesical bacteriophages; however, these therapies are very much in their infancy and limited to in-vitro studies [8].

Glycosaminoglycan instillations

In rUTIs, damage to the bladder's protective GAG layer makes the urothelium more vulnerable to bacterial adherence and colonisation. This compromised barrier allows bacteria to attach to the bladder wall, leading to repeated infections. The damaged GAG layer also increases bladder permeability, which can exacerbate inflammation and contribute to persistent infection cycles [9].

Glycosaminoglycan instillations aim to restore this protective barrier by replenishing the depleted GAG molecules, such as hyaluronic acid or chondroitin sulphate. By reinforcing the urothelial lining, GAG instillations prevent bacterial adhesion, reduce inflammation, and lower bladder permeability. This approach not only helps protect the bladder from future infections but also interrupts the cycle of rUTIs by promoting healing of the bladder lining and creating an environment less conducive to bacterial colonisation [9].

Intravesical antibiotics

In rUTIs, bacterial pathogens colonise the bladder urothelium or form biofilms, making it difficult for systemic antibiotics to eradicate them fully, leading to cycles of inflammation and tissue damage and ultimately resulting in chronic infections. Instillation of antibiotics like gentamicin, amikacin, or ciprofloxacin allows high local concentrations to develop directly at the infection site, bypassing issues of poor antibiotic penetration into the bladder from systemic circulation [10].

Certain antibiotics penetrate and disrupt bacterial biofilms, which are protective structures that harbour bacteria and contribute to antibiotic resistance. Intravesical antibiotics can more effectively reach and kill bacteria entrenched in these biofilms within the bladder. As the antibiotics are delivered directly to the infection site at high concentrations, they can be more effective against resistant bacteria. This approach can reduce the selective pressure on other microbial flora in the body, potentially minimising the development of antibiotic resistance [11].

## Review

Methods

Search Strategy

A systematic review was undertaken in adherence to the Preferred Reporting for Systematic Reviews and Meta-Analyses (PRISMA) [12]. A computer-assisted search was undertaken of the PubMed and Cochrane Controlled Register of Trials (CENTRAL) databases on August 3, 2024. The search included keywords and medical subject headings “intravesical” and “UTI” or “urinary tract infection” and only included articles from 2010 onwards.

Study Selection

This review included only peer-reviewed articles, available in English. The population of interest was limited to adults, with a definition of rUTI being uncomplicated UTIs with a frequency of two in six months or three within a year, as per the European Association of Urology definition [13]. Studies involving patients with known structural abnormalities of the urinary tract or neurogenic bladder dysfunction were excluded. The intervention needed to be an intravesical therapy used for prophylaxis of rUTIs. The primary outcome was the rate of UTI or the number of UTIs after treatment; however, a secondary outcome looked at tolerability and side effects relating to intravesical therapy. In-vitro studies and in-vivo studies in animals were excluded.

Literature Screening

**Figure 1:**
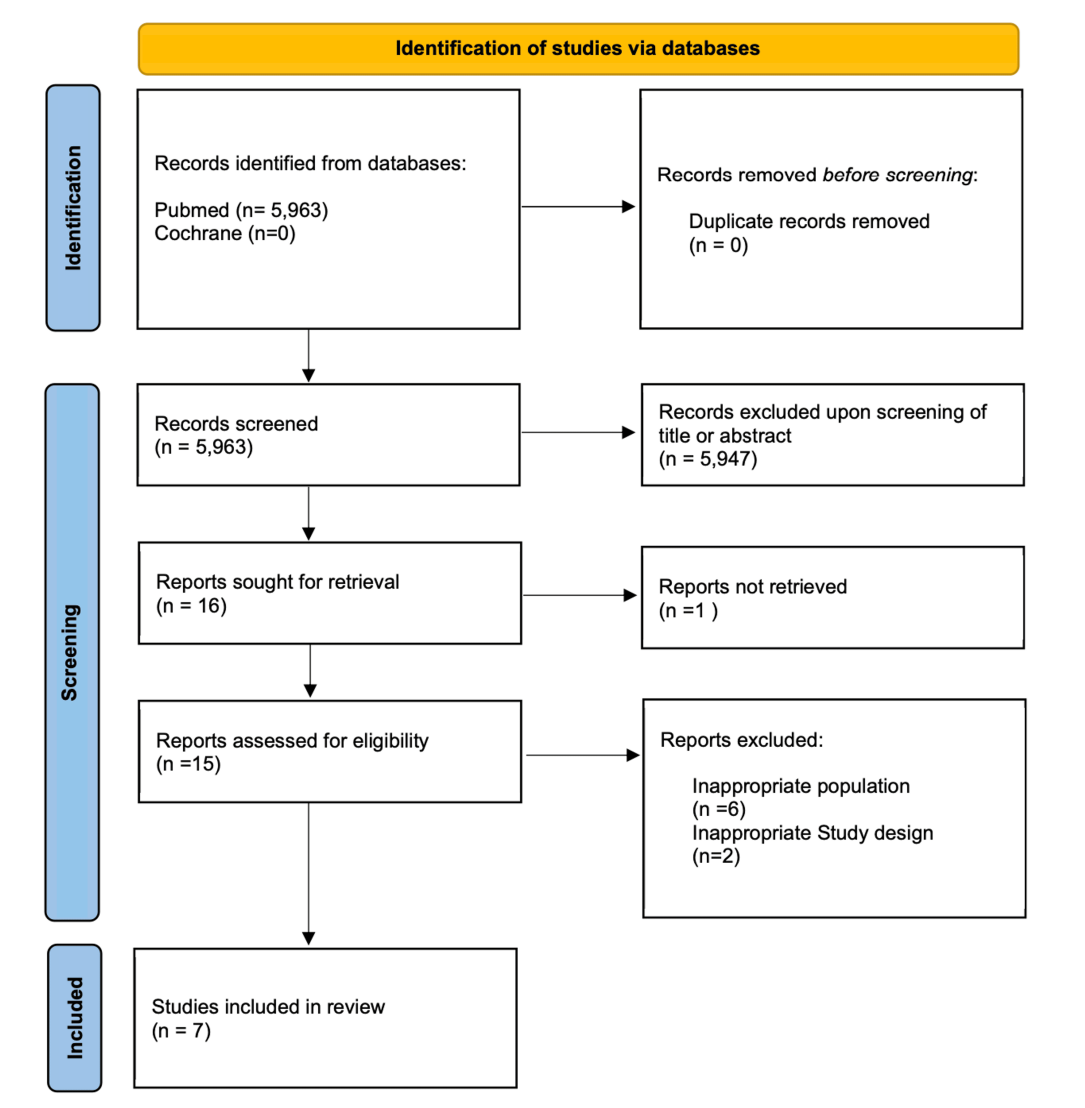
A PRISMA flowchart of the literature search and screening process PRISMA: Preferred Reporting Items for Systematic Reviews and Meta-Analyses

The initial literature search yielded 5,963 reports. The titles and abstracts were screened independently, and inappropriate studies were filtered out at this stage. Sixteen records were sought for retrieval, but one was not available in full-text format. Fifteen records were scrutinised to determine if they met the inclusion and exclusion criteria, leading to seven papers being included in the final review. A Preferred Reporting Items for Systematic Reviews and Meta-Analyses (PRISMA) flowchart of the literature search and screening is shown in Figure 1 [12].

Results

Of the seven studies included, two were meta-analyses, two were retrospective cohort studies, two were case series, and one was a case-control study. The year of publication spanned 2012 to 2022, and the population size within each study varied greatly. Table 1 provides an overview of the included studies.

**Table 1:** An overview of the included studies

Author and year	Sample size	Population	Study Type	Intervention	Results
Batura et al., 2019 [14]	N=18	Adult females with recurrent urinary tract infections (rUTIs) who have trialled preventative therapy	Retrospective cohort study	Hyaluronic acid (HA) (Cystistat®), varying doses (40 mg or 120 mg), varying schedules (one to 14 instillations in total).	Significant reduction in urinary tract infections (UTIs) per year from a median of 10 to two. Significant reduction in pain, frequency, nocturia, urgency, and loss of sleep Improvement in quality of life but not statistically significant. Most patients got a burning sensation during instillation.
Cicione et al., 2014 [15]	N=157	Adult females with rUTIs. Patients were excluded if any known bladder pathology, high post-void residual or prophylactic antibiotics were noted.	Retrospective cohort study	HA + chondroitin sulphate (CS) (Ialuril®); weekly instillation for four weeks then once a month for five months.	Significant reduction in UTIs per year from 4.1 to 0.4. Significantly longer time to UTI recurrence from 95 days to 178 days. Significantly improved pain/frequency/symptom scores in the first 12 months but then returned to baseline at 24 months. Ten patients reported storage urinary symptoms, of which one required medication.
De Vita et al., 2012 [16]	N=143	Four studies; all had a population of adult females with rUTIs	Meta-analysis	Two studies with Cystistat® and two studies with iAluRil®	Significantly reduced number of UTIs per year in all four studies. Significantly longer time to UTI recurrence in all four studies. No change in urinary frequency in the HA+CS group (not recorded in the HA-only group)
Goddard and Janssen, 2017 [17]	N=800	Eight studies; all had a population of adult females with rUTIs	Systematic review and meta-analysis	Five studies with iAluRil® and three studies with Cystistat®	Overall significant reduction in the number of UTIs per year by 2.6. Overall significant increase in time to recurrence by 130 days. Overall significant improvement in pain and urinary urgency and frequency symptom score by 6.5; 20% of cases reported mild irritation during instillation.
Gugliotta et al., 2015 [18]	N=174	Adult females with rUTIs	Case-control study	iAluRil®; once a week for one month then monthly for four months	A significantly higher number of patients were UTI-free after one year in the iAluRil® group vs. the control group. Significantly more likely to get UTI recurrence in the control group vs. the iAluRil® group; 22% of the iAluRil® group reported burning during instillation.
Chernyak and Salamon 2020 [19]	N =12	Post-menopausal females with rUTIs	Case series	Intravesical gentamicin 80 mg was instilled twice per week for three weeks (tobramycin 80 mg was used in one patient with gentamicin resistance)	Significantly reduced the number of UTIs after instillations. No antibiotic-resistant pathogens were found in urine after six months. All treatment courses completed with good tolerability
Harte, Magee, and De Barra, 2022 [20]	N =3	rUTIs in immunosuppressed patients (one male, two female patients); all had previous cystoscopy and urodynamics to ensure no anatomical abnormality	Case series	Intravesical amikacin daily for two weeks then alternate days for 10 weeks then twice per week for 12 weeks	Successful resolution of symptoms in two of three patients. All patients tolerated instillations with no side effects. All had undetectable serum aminoglycoside levels throughout the schedule. All patients reported improved quality of life.

Discussion

This systematic review has demonstrated the paucity of high-quality data in this particular field within the chosen population. Unfortunately, several studies that may have held relevance required exclusion due to the cohort containing patients with neurogenic bladder dysfunction. This was important to avoid skewed tolerability ratings based on the need for intermittent self-catheterisation (ISC) in intravesical therapy, as many patients with neurogenic bladder dysfunction either already carry out ISC or have an indwelling catheter [7]. It has been observed that ISC can be a huge barrier to being able to initiate and tolerate intravesical instillations, whereby compliance rates as low as 58% at one year have been reported [21]. The most commonly reported reasons for poor compliance include discomfort, fear of injury, lack of education, embarrassment, and difficulties with dexterity. 

Overall, every study in this review demonstrated with statistical significance that intravesical GAG and intravesical antibiotic installations both reduce UTI recurrence rates. In all studies, there were no reported serious adverse events associated with both types of intravesical therapy and no reports of dropouts due to inability to tolerate the instillations [14-20]. However, it must be mentioned that GAG instillations were reported to cause burning or irritation of the bladder during and immediately after instillations. Most patients did not require any treatment for this and described it as brief and self-limiting, but a very small proportion of patients required anti-inflammatory drugs for this side effect [15]. The two studies assessing intravesical antibiotics did not report any side effects, but the population sizes in both studies were small [19,20], further highlighting the lack of evidence available for intravesical antibiotic use in non-neuropathic patients.

Another issue highlighted by this review is the variation in schedules for each intravesical therapy. For GAG instillations, the generic consensus amongst all studies was to have an “induction course” where instillations were given weekly for one month, followed by a less intensive “maintenance” schedule. Theoretically, a duration of at least three months of instillations is required to create a long-term change in the GAG layer [22]. However, the reported regularity of maintenance installations differed within the studies analysed. Some studies defined maintenance with a frequency of monthly installations, while others used fortnightly, and the total duration of delivery of maintenance therapy varied greatly from four weeks to seven months. The most common schedule was an induction course followed by four months of maintenance, but there is a lack of robust data to support this as the most effective schedule. Similarly, there were varying doses and schedules in the intravesical antibiotic studies, with no universal consensus on the total duration of treatment nor the frequency.

In this review, only Goddard and Janssen [17] and De Vita et al. [16] included studies that involved different types of GAG instillation. Both meta-analyses included studies evaluating both hyaluronic acid and chondroitin sulphate (iAluRil®) and hyaluronic acid alone (Cystistat®). Both studies found that each therapy significantly reduced the recurrence rate of UTIs, but randomised controlled trials were only available for iAluRil®. Since no randomised controlled trials exist for Cystistat®, the authors were not able to make statistical comparisons between the two therapies and therefore concluded that while both have been shown to reduce UTI rates, iAluRil® has greater supportive evidence. Similarly, there are no available studies comparing the effect of GAG instillations versus intravesical antibiotics to determine which therapy may be superior in treating rUTIs, so clinicians are unable to give an evidence-based recommendation over which intravesical therapy is most suitable for their patients.

Chernyak and Salamon [19] proved to be the only study to take serial urine cultures during the course of instillations; the other studies took urine cultures only when patients self-presented with symptoms of UTI. During their six-month follow-up protocol, not one patient grew an antibiotic-resistant pathogen. This finding suggests that intravesical antibiotics are an excellent alternative in terms of reducing levels of multi-resistant bacteria. In comparison, 20% of adults on continual antibiotic prophylaxis for more than a year have been found to develop antimicrobial-resistant uropathogens [23]. Despite Chernyak and Salamon’s promising findings in relation to antimicrobial resistance, they are limited by the fact they had a very small sample size (n = 17) and only followed up with patients for six months.

Another factor that cannot be ignored is the cost associated with intravesical therapy. The total cost per annum of the most common regime of iAluRil® is £704, versus £12 for trimethoprim prophylaxis [24]. Additionally, there are hidden costs associated with intravesical instillations, such as ISC equipment, the cost of hosting clinics for ISC teaching, and regular serum gentamicin monitoring for those on intravesical gentamicin. On the other hand, the cost of running a hospital bed in the United Kingdom for one day is £586 [25]. Therefore, if the proposed therapies successfully reduced hospital stays by one to two days per patient per year, they could be cost-saving. Further studies exploring a cost analysis of intravesical therapies are required to see their overall financial impact.

## Conclusions

Intravesical therapies, as a whole, have been shown to significantly reduce UTI recurrence rates, improve symptom scores, and are very well tolerated in patients without neurogenic bladder dysfunction. In particular, intravesical antibiotics have shown great promise in minimising antimicrobial resistance in those patients who have previously grown multi-resistant uropathogens; however, further studies with larger population sizes and a greater length of follow-up are required before a definite conclusion can be drawn.

Further randomised controlled studies are required to determine the efficacy of intravesical antibiotics and different forms of GAG instillation independently, with subsequent systematic reviews or meta-analyses to allow for comparisons on which form of therapy has the greater benefit for patients. While costs are a potential issue, further studies evaluating the impact of these therapies on the number of hospital days or admissions would help in clarifying their net financial benefit and aid subsequent integration into public health settings. 
